# Atomic-Layer-Deposition of Indium Oxide Nano-films for Thin-Film Transistors

**DOI:** 10.1186/s11671-017-2414-0

**Published:** 2018-01-09

**Authors:** Qian Ma, He-Mei Zheng, Yan Shao, Bao Zhu, Wen-Jun Liu, Shi-Jin Ding, David Wei Zhang

**Affiliations:** State Key Laboratory of ASIC and System, Shanghai Institute of Intelligent Electronics and Systems, School of Microelectronics, Fudan University, Shanghai, 200433 China

**Keywords:** Atomic layer deposition, Low deposition temperature, In_2_O_3_, Thin-film transistors

## Abstract

Atomic-layer-deposition (ALD) of In_2_O_3_ nano-films has been investigated using cyclopentadienyl indium (InCp) and hydrogen peroxide (H_2_O_2_) as precursors. The In_2_O_3_ films can be deposited preferentially at relatively low temperatures of 160–200 °C, exhibiting a stable growth rate of 1.4–1.5 Å/cycle. The surface roughness of the deposited film increases gradually with deposition temperature, which is attributed to the enhanced crystallization of the film at a higher deposition temperature. As the deposition temperature increases from 150 to 200 °C, the optical band gap (E_g_) of the deposited film rises from 3.42 to 3.75 eV. In addition, with the increase of deposition temperature, the atomic ratio of In to O in the as-deposited film gradually shifts towards that in the stoichiometric In_2_O_3_, and the carbon content also reduces by degrees. For 200 °C deposition temperature, the deposited film exhibits an In:O ratio of 1:1.36 and no carbon incorporation. Further, high-performance In_2_O_3_ thin-film transistors with an Al_2_O_3_ gate dielectric were achieved by post-annealing in air at 300 °C for appropriate time, demonstrating a field-effect mobility of 7.8 cm^2^/V⋅s, a subthreshold swing of 0.32 V/dec, and an on/off current ratio of 10^7^. This was ascribed to passivation of oxygen vacancies in the device channel.

## Background

Indium oxide (In_2_O_3_) is a transparent metal oxide semiconductor, which exhibits a wide band gap of ~3.7 eV at room temperature, a high transparency for visible light, and excellent chemical stability [1, 2]. Therefore, In_2_O_3_ has been investigated for various applications such as photovoltaic devices, electrochemical sensors, and flat panel displays [3–5]. So far, several deposition techniques have been developed to prepare In_2_O_3_ thin-films, including sputtering [6, 7], sol-gel [8, 9], and chemical vapor deposition (CVD) [10, 11]. However, the techniques of sputtering and sol-gel usually suffer from a poor uniformity across a large area as well as inexact elemental composition; the CVD technique generally requires relatively high deposition temperatures of > 300 °C. These drawbacks make it challenging to achieve a uniform In_2_O_3_ film with precise thickness and composition control at a low deposition temperature.

In recent years, atomic-layer-deposition (ALD) has emerged as a promising approach that can yield excellent step coverage, atomic scale thickness controllability, good uniformity, and a relatively low deposition temperature. Accordingly, the growth of In_2_O_3_ thin-films has been explored by means of ALD with different precursors, including InCl_3_-H_2_O [12], InCl_3_-H_2_O_2_ [13], InCp-O_3_ [14], InCp-O_2_-H_2_O [15], and In (CH_3_)_3_-H_2_O [16]. In terms of the precursors of InCl_3_-H_2_O and InCl_3_-H_2_O_2_, the deposition temperatures for In_2_O_3_ films must be increased to ~ 300–500 °C [13]; meanwhile, the InCl_3_ container should be heated to 285 °C in order to obtain ample InCl_3_ vapor [15]. Furthermore, the byproduct of corrosive HCl can damage the ALD equipment and etch the deposited In_2_O_3_ film [17], and the growth rate of In_2_O_3_ is as low as 0.25–0.40 Å/cycle. Although other precursors such as TMIn-H_2_O and TMIn-H_2_O_2_ have been adopted for ALD In_2_O_3_ films, the deposition temperatures are still high (i.e., 200–450 °C) in spite of relatively large growth rates (1.3–2 Å/cycle) [18].

In this work, InCp and H_2_O_2_ were proposed as the precursors of ALD In_2_O_3_ thin-films, thus the In_2_O_3_ thin-films were deposited successfully at lower temperatures, exhibiting a satisfactory growth rate. Additionally, the physical and chemical properties of the deposited films were characterized. Further, the In_2_O_3_ thin-film transistors (TFTs) with ALD Al_2_O_3_ gate dielectrics have been fabricated, demonstrating good electrical performance, such as a field effect mobility of 7.8 cm^2^ V^−1^ s^−1^, and an on/off current ratio of 10^7^ etc.

## Experimental

Si (100) wafers were cleaned using the standard Radio Corporation of America process, serving as the initial substrates. In_2_O_3_ thin-films were deposited onto the pre-cleaned Si (100) substrates using the ALD equipment (Wuxi MNT Micro Nanotech Co., LTD, China) at relatively low temperatures of 150–210 °C, where the temperatures of InCp (Fornano Electronic Technology Co., LTD, China, impurity: 99.999%) and H_2_O_2_ (30% aqueous solution) precursors were maintained at 130 and 50 °C, respectively. Nitrogen gas was used as a purging gas. To demonstrate the function of the ALD In_2_O_3_ thin-film, the In_2_O_3_-based channel TFTs were fabricated as the following processes. Firstly, a 38-nm Al_2_O_3_ gate dielectric film was grown on a pre-cleaned p-type Si (100) substrate (< 0.0015 Ω·cm) at 200 °C by ALD using trimethylaluminium and H_2_O, and such low resistivity silicon substrate served as the back gate. Then, a 20-nm In_2_O_3_ channel layer was grown on the Al_2_O_3_ film at 160 °C. Source/drain contacts of Ti/Au (30 nm/70 nm) stacks were formed in turn by optical lithography, electron beam evaporation and a lift-off process. Finally, the fabricated devices were annealed at 300 °C in air for different times.

The crystallinity, surface morphology, elemental composition, absorption coefficient, and thickness of the In_2_O_3_ films were characterized by X-ray diffraction (XRD) (Bruker D8 Discover), atomic force microscopy (AFM) (Bruker Icon), X-ray photoelectron spectroscopy (XPS) (Kratos Axis Ultra DLD), ultraviolet-visible spectroscopy (UV-VIS), and ellipsometer (Sopra GES-SE, France), respectively. The electrical measurements of the devices were performed using a semiconductor parameter analyzer (B1500A, Agilent Technologies, Japan) with Cascade probe station in ambient air at room temperature.

## Results and Discussion

**Fig. 1:**
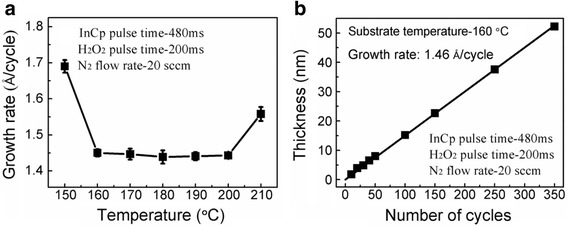
**a** Growth rate of ALD In_2_O_3_ film on the Si substrate as a function of substrate temperature, and **b** dependence of the In_2_O_3_ film thickness on the number of ALD cycles at 160 °C

Figure 1a shows the growth rate of the In_2_O_3_ film as a function of substrate temperature. It is found that a stable growth rate of ~ 1.46 Å/cycle is achieved in the range of 160 ~ 200 °C, revealing a fast growth rate and a well-defined temperature window for ALD In_2_O_3_ films. When the substrate temperature was reduced to 150 °C or increased to 210 °C, the resulting growth rate became larger [19, 20]. The former is attributed to the condensation of InCp on the substrate, whereas the latter is due to the thermal decomposition of InCp at a higher temperature. Further, the evolution of the deposited In_2_O_3_ film thickness was evaluated as a function of ALD cycles, as shown in Fig. 1b. It is clear that the In_2_O_3_ film thickness increases linearly with the number of deposition cycles, indicative of quite uniform growth.

**Fig. 2:**
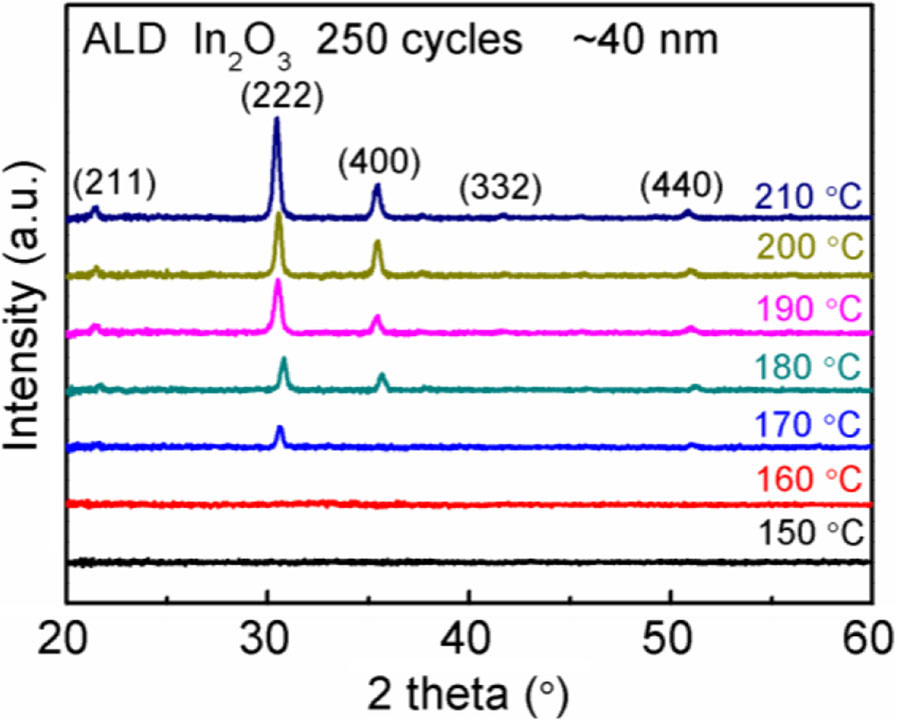
X-ray diffraction patterns of the In_2_O_3_ films deposited at different temperatures for 250 cycles

**Fig. 3:**
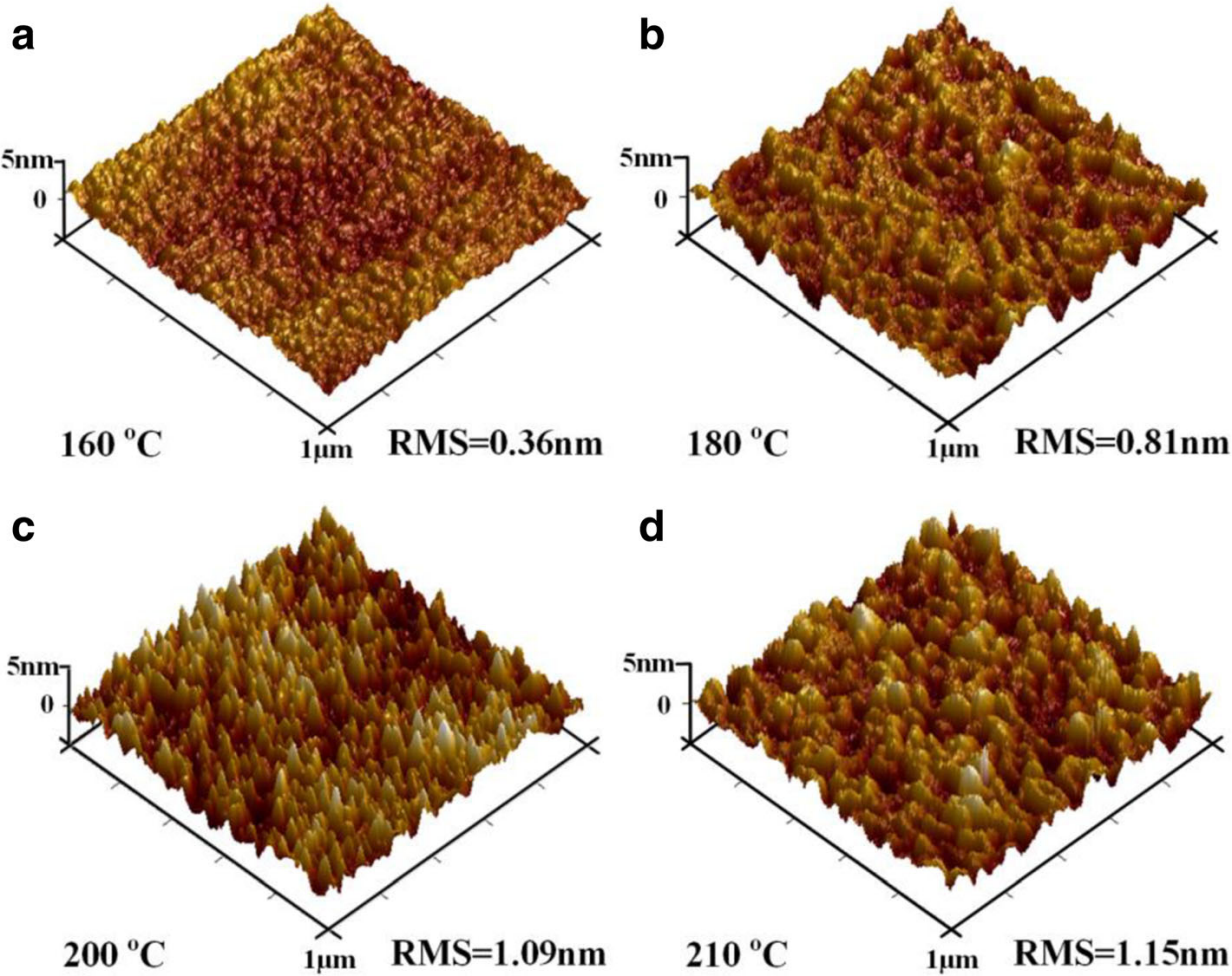
The AFM images of the In_2_O_3_ films deposited at different temperatures: **a** 160 °C, **b** 180 °C, **c** 200 °C and **d** 210 °C. The deposition cycles were fixed at 250 for each film

To observe the evolution of the In_2_O_3_ film texture with deposition temperature, the XRD patterns of the In_2_O_3_ films deposited at different temperatures are presented in Fig. 2. When the deposition temperature does not exceed 160 °C, no diffraction peak can be observed. This indicates that the deposited In_2_O_3_ films at lower temperatures are amorphous. When the deposition temperature increases up to 170 °C, some diffraction peaks start to appear. Further, with the deposition temperature gradually increasing to 210 °C, the diffraction peak intensities increase dramatically, typically shown by the peaks at 2θ = 30.3° and 35.4°. This indicates that the crystallinity and grain size of the as-deposited In_2_O_3_ film are enhanced gradually with increasing the deposition temperature. Figure 3 shows the surface morphologies of the representative In_2_O_3_ films deposited at different temperatures. It is found that the film surface becomes rougher and rougher with increasing the deposition temperature, i.e., the resulting root-mean-square (RMS) roughness increases from 0.36 to 1.15 nm with increasing the deposition temperature from 160 to 210 °C. This should be related to the crystallinity of the In_2_O_3_ film. In terms of the deposition temperature of 160 °C, the deposited In_2_O_3_ film is amorphous, and it exhibits a very smooth surface. When the deposition temperature attains 180 °C, the deposited film becomes polycrystalline. This means that the resulting film contains lots of crystalline grains, and the grain sizes become larger and larger with increasing the deposition temperature, as revealed in Fig. 2. This is in good agreement with our observation that the sizes of hillocks on the film surface gradually increase with raising the deposition temperature, hence resulting in an increased RMS value.

**Fig. 4:**
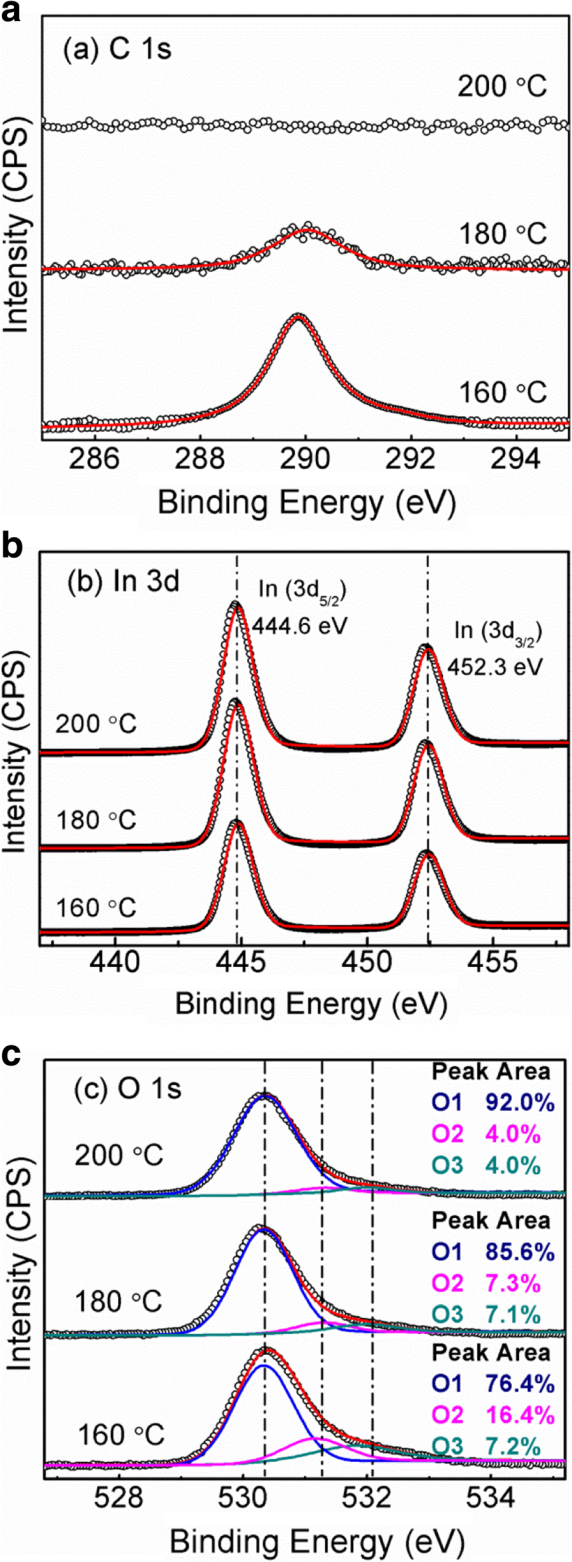
High resolution **a** C 1s, **b** In 3d, and **c** O 1 s XPS spectra of the In_2_O_3_ films deposited at 160, 180, and 200 °C, respectively. To remove adventitious surface contaminants, all the samples were etched with in-situ Ar ion bombardment for 6 min before signal collection

Figure 4 shows high resolution C 1 s, In 3d and O1s XPS spectra of the In_2_O_3_ films deposited at different temperatures. Regarding the C 1 s XPS spectra shown in Fig. 4a, the film deposited at 160 °C displays a peak at 289.8 eV, which should correspond to C-O [21]. When the deposition temperature is increased to 180 °C, the peak becomes much weaker. Further, in terms of 200 °C deposition temperature, the C 1 s peak disappears. Thus, it is indicated that the higher the deposition temperature, the less the impurity of C in the deposited In_2_O_3_ film. Figure 4b depicts the In 3d XPS spectra of the In_2_O_3_ films, clearly demonstrating one-doublet Gaussian peaks at 444.7 and 452.3 eV, which are associated with In 3d_5/2_ and In 3d_5/2_ core levels for In_2_O_3_ [22, 23]. The O 1 s XPS spectra are shown in Fig. 4c. It is found that the O 1 s spectrum for each sample can be well separated into three peaks, which are located at 529.8, 531.0, and 532.0 eV, respectively. These peaks correspond to O^2−^ ions bound with metal (O1), oxygen vacancies (O2) and –OH/CO (O3), respectively [24, 25]. As the deposition temperature increases from 160 to 200 °C, the relative percentage of O1 increases from 76 to 92%; and the relative percentage of O2 decreases gradually from 16 to 4%. Moreover, the relative percentage of O3 also exhibits a downward trend. These results indicate that a higher deposition temperature is beneficial to reduce the concentration of oxygen vacancies in the deposited film as well as hydroxyl groups and C−O bonds. Further, the elemental compositions of the In_2_O_3_ films deposited at different temperatures are listed in Table 1. Interestingly, the atomic ratio of In/O in the deposited film decreases by degrees with increasing deposition temperature. However, even for the pure In_2_O_3_ film deposited at 200 °C, the atomic ratio (1:1.36) of In/O is still larger than that (1:1.5) of the stoichiometric In_2_O_3_. This reveals that the ALD In_2_O_3_ film is generally rich in oxygen vacancies.

**Table 1:** The Elemental Percentages of In_2_O_3_ Films Deposited at Different Temperatures

Deposition Temperature	C	In	O	Atomic Ratio of In:O
160 °C	6.9%	42.9%	50.2%	1:1.17
180 °C	1.1%	42.3%	56.5%	1:1.34
200 °C	0	42.3%	57.7%	1:1.36

**Fig. 5:**
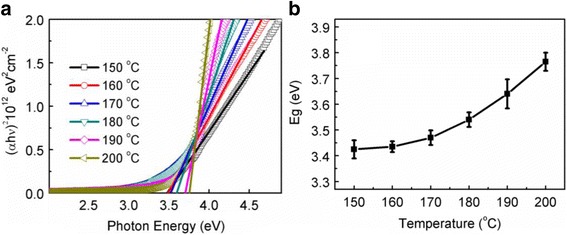
**a** Plotting of (αhν)^2^ vs photon energy for the In_2_O_3_ films deposited at different temperatures; **b** dependence of the extracted band gap (E_g_) of In_2_O_3_ on deposition temperature

Figure 5a shows the variation of (αhν)^2^ as a function of photon energy for the as-deposited In_2_O_3_ films at different deposition temperatures. The optical band gap (E_g_) of the In_2_O_3_ film can be determined by the Tauc’s relation: αhν = A(hν-E_g_)^n^ [26], where *α* is the absorption coefficient, *A* is a constant, *h* is the Plank constant, *ν* is the frequency, and the exponent *n* characterizes the nature of band transition. Here, *n* = 1/2, indicating that In_2_O_3_ is a semiconductor with a directly allowed transition. *E*_g_ is extracted by extrapolating the straight line portion to the energy bias at *α* = 0. The extracted *E*_g_ for the In_2_O_3_ film is shown in Fig. 5b. It is seen that *E*_g_ increases from 3.42 to 3.75 eV with raising the deposition temperature from 150 to 200 °C. The increased *E*_g_ at higher deposition temperatures could result from the reductions of oxygen vacancies and C impurity in the deposited film. In fact, other researchers also reported that when lots of oxygen vacancies existed in ZnO, the impurities states became more delocalized and overlapped with the valence band edge, thus leading to the band gap narrowing [27]. In addition, the gradually enhanced crystallinity as a function of deposition temperature could influence the optical band gap of the In_2_O_3_ film. This can be explained as follows. As the deposition temperature rises, the grain size of the deposited In_2_O_3_ film increases, shown in Fig. 2. This thus leads to a decrease in the density of grain boundaries in the film. Since electrons are easily trapped in the grain boundaries, the number of free electrons should increase in the In_2_O_3_ film with less grain boundaries [28, 29]. Therefore, such an increased electron concentration results in a larger optical band gap due to the Burstein-Moss shift [30].

**Fig. 6:**
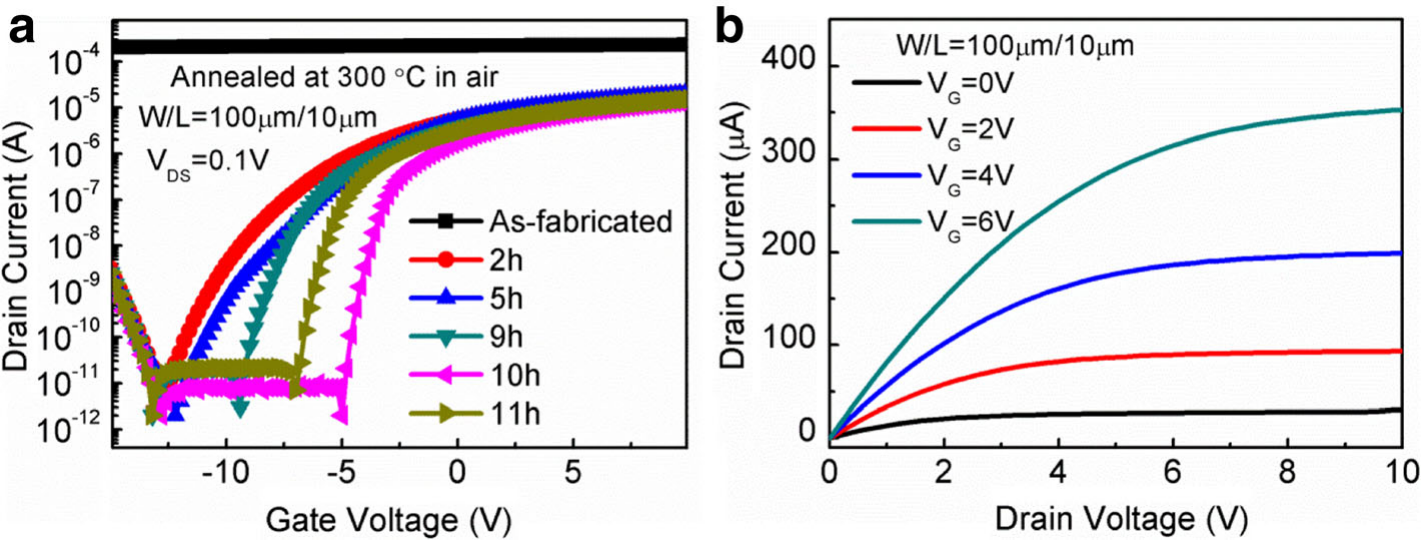
**a** Transfer characteristics of the In_2_O_3_ TFTs annealed at 300 °C in air for different time; **b** Output characteristics of the In_2_O_3_ TFT annealed at 300 °C in air for 10 h

To demonstrate the function of the ALD In_2_O_3_ film acting as the channel of TFT, the In_2_O_3_-channel-based TFTs with atomic-layer-deposited Al_2_O_3_ gate dielectrics were fabricated. Figure 6a shows the transfer characteristics of In_2_O_3_ TFTs. It is found that the as-fabricated device does not exhibit the switch characteristics typical of field-effect transistors, but a conductor-like between source and drain. This should be attributed to the existence of lots of oxygen vacancies in the In_2_O_3_ channel because oxygen vacancies can supply free electrons. Therefore, for the sake of reducing the concentration of oxygen vacancies in the In_2_O_3_ channel, post-annealing in air was carried out at 300 °C. It is clear that the In_2_O_3_ TFT exhibits a typical switching behavior after 2-h annealing. This indicates that the post-annealing in air can improve significantly the device performance. Further, as the annealing time increases gradually to 10 h, the threshold voltage (V_th_) of the TFT shifts in the direction of positive bias, and the sub-threshold swing (SS) improves little by little. However, when the annealing time increases to 11 h, the device performance starts to degenerate. It is noted that hydrogen may be incorporated into the film during the fabrication process, acting as an electron trap by forming –OH bonds in the channel or at the interface between the channel and dielectric [31]. These electron traps perhaps result in the degradation of SS. After annealing in air, the OH bonds were reduced by incorporation of O_2_ molecules [32]. This could lead to a decrease in the trap density, thus improving the SS of the device. In terms of 10 h annealing in air, the In_2_O_3_ TFT exhibits a field-effect mobility (μ_EF_) of 7.8 cm^2^ V^−1^ s^−1^, a V_th_ of −3.7 V, a SS of 0.32 V/dec, and an on/off current ratio (I_on_/I_off_) of 10^7^. The corresponding output characteristics are also presented in Fig. 6b, demonstrating clear pinch-off and current saturation behaviors under various positive gate voltages. Furthermore, the output curves also indicate an *n*-type enhancement mode. For comparison, Table 2 summarizes the characteristics of the reported ALD In_2_O_3_ films and TFTs from different research groups [33–37]. It is demonstrated that our In_2_O_3_ film shows a superior growth rate at a relatively low temperature, and the fabricated device also exhibits a small SS. However, the general performance of the device is not so perfect, which could be improved via some process and device structure optimizations.

**Table 2:** Characteristics of the ALD In_2_O_3_ Films and In_2_O_3_ TFTs From Different Groups

Oxygen Precursor	Metal Precursor	Deposition Temperature (°C)	Growth rate (Å/cycle)	Channel Thickness (nm)	μ (cm^2^/V·s)	V_TH_ (V)	I_ON_/I_OFF_	SS (V/dec)	Ref.
H_2_O	Et_2_InN(TMS)_2_^a^	175~250	0.7	–	–	–	–	–	[23]
H_2_O	DMLDMIn^b^	300~350	0.6	–	–	–	–	–	[33]
O_2_ plasma	Et_2_InN(SiMe_3_)_2_^c^	250	1.45	5	39.2	−1.18	–	0.27	[34]
H_2_O	TMIn^d^	200~251	0.39	–	–	–	–	–	[35]
H_2_O_2_	InCA-1^e^	125	0.6	18	15	−0.2	10^8^	–	[36]
H_2_O_2_	InCA-1^e^	150	0.6	18	9.8	−0.2	10^9^	0.63	[37]
H_2_O_2_	InCp	160	1.46	20	7.8	−3.7	10^7^	0.32	This work

^a^Et_2_InN(TMS)_2_ represents the diethyl [bis (trimethylsilyl) amido]- indium

^b^DMLDMIn represents the dimethylamino-dimethylindium

^c^Et_2_InN(SiMe_3_)_2_ represents the diethyl [bis (trimethylsilyl) amido] indium

^d^TMIn represents the trimethyl indium

^e^InCA-1 represents the [1,1,1-trimethyl-N-(trimethylsilyl) silanaminato]-Indium

To well understand the influence of post-annealing in air on the composition of the In_2_O_3_ channel, the In_2_O_3_ films were annealed at 300 °C for different times, and then were analyzed by means of XPS. Table 3 lists the elemental percentages of various annealed films. As the annealing time increases from 2 to 11 h, the atomic ratio of In:O decreases from 1:1.22 to 1:1.48, gradually approaching that (1:1.5) of the stoichiometric In_2_O_3_. This further confirms that increasing annealing time in air effectively reduced the density of oxygen vacancies in the In_2_O_3_ film. Therefore, the improvement in the performance of the In_2_O_3_ TFT should be mainly attributed to the passivation of oxygen vacancies which could be located in the bulk channel and/or the interface between the channel and the dielectric [25]. However, the excessive annealing degraded the performance of the device, as revealed by 11 h annealing. This could be ascribed to the change of crystallization of the In_2_O_3_ channel layer as well as possible oxidation of Ti electrodes during superfluous post-annealing in air. Thus, an appropriate annealing time is required in order to achieve good performance of the In_2_O_3_ TFT.

**Table 3:** The Elemental Percentages of In_2_O_3_ Films Annealed at 300 °C in Air for Different Time

Annealing time	C	In	O	In:O
2 h	5.9%	42.4%	51.7%	1:1.22
7 h	6.2%	41.6%	52.2%	1:1.25
10 h	6.3%	39.8%	53.9%	1:1.35
11 h	4.8%	38.4%	56.8%	1:1.48

All the films were deposited at 160 °C and etched with in-situ Ar ion bombardment

## Conclusions

The fast ALD growth of the In_2_O_3_ films has been achieved at relatively low temperatures (160–200 °C) with the InCp and H_2_O_2_ precursors, exhibiting a uniform growth rate of 1.46 Å/cycle. As the deposition temperature increased, the crystallization of the deposited film was enhanced gradually. Meanwhile, both oxygen vacancies and carbon impurities in the deposited films were also reduced significantly. This thus led to an increase in the *E*_g_ of In_2_O_3_. Further, with the ALD In_2_O_3_ channel layer, the TFTs with an ALD Al_2_O_3_ dielectric were fabricated. As the post-annealing time in air was lengthened, the electrical performance of the In_2_O_3_ TFT was improved distinctly till 10 h annealing. This is mainly due to the passivation of oxygen vacancies located in the bulk channel and/or the interface between the channel and the dielectric after annealing in air. In terms of 10 h annealing, the device exhibited good performance such as a field-effect mobility of 7.8 cm^2^/V⋅s, a subthreshold swing of 0.32 V/dec, and an on/off current ratio of 10^7^. In terms of 200 °C deposition temperature, the deposited film exhibits an In:O ratio of 1:1.36 without detectable carbon, thus revealing the existence of oxygen vacancies in the as-deposited film.
